# Paraneoplastic subacute cutaneous lupus erythematosus with mucositis

**DOI:** 10.1016/j.jdcr.2022.02.001

**Published:** 2022-02-17

**Authors:** James Abbott, J. Skylar Westerdahl, David Wada, Stephanie Klein, Jason Mathis

**Affiliations:** a Department of Dermatology, University of Utah, Salt Lake City, Utah; b University of Utah School of Medicine, Salt Lake City, Utah

**Keywords:** non–small cell lung carcinoma, paraneoplastic, SCLE, subacute cutaneous lupus erythematosus, ANA, antinuclear antibodies, IgG, immunoglobulin G, SCLE, subacute cutaneous lupus erythematosus

## Introduction

Subacute cutaneous lupus erythematosus (SCLE) is a clinical phenotype of cutaneous lupus erythematosus first described by Sontheimer et al^1^ in 1979.This entity demonstrates characteristic clinical and serologic findings, with patients commonly developing nonscarring annular-to-arcuate polycyclic papulosquamous plaques on sun-exposed skin in the setting of mild systemic manifestations and the presence of Ro/Sjogren's-syndrome-related antigen (SSA) autoantibodies.

Classically, SCLE has been associated with drug-related triggers; however, SCLE has also presented as a paraneoplastic phenomenon related to internal malignancies.

Here, we report a case of paraneoplastic SCLE with extensive mucositis due to small-cell lung carcinoma.

## Case report

A 62-year-old cachectic woman with a 40-year smoking history, hypertension, diabetes mellitus type 2, and gastroesophageal reflux disease presented to dermatology clinic with a 3-month history of painful oral erosions and ulcerations affecting her gingiva, tongue, and oral mucosa. The patient initially developed white stuck-on lesions that became progressively more painful over 1 to 2 months, leading to erosions. Since the onset of the erosions, she had increasingly limited her oral intake, and at the time of the visit could only tolerate thick liquids. Additionally, the patient reported developing a pruritic rash involving her upper extremities, chest, and back following the same timeframe. She had tried both medium-potency topical steroids and various over-the-counter products without relief. She recently had been fitted for new dentures; however, she was unable to use them due to severe mouth pain. Otherwise, she denied any new occupational or social exposures or medications in the past 6 months. Her review of systems was positive for increased fatigue, weight loss, and a chronic cough.

**Fig 1:**
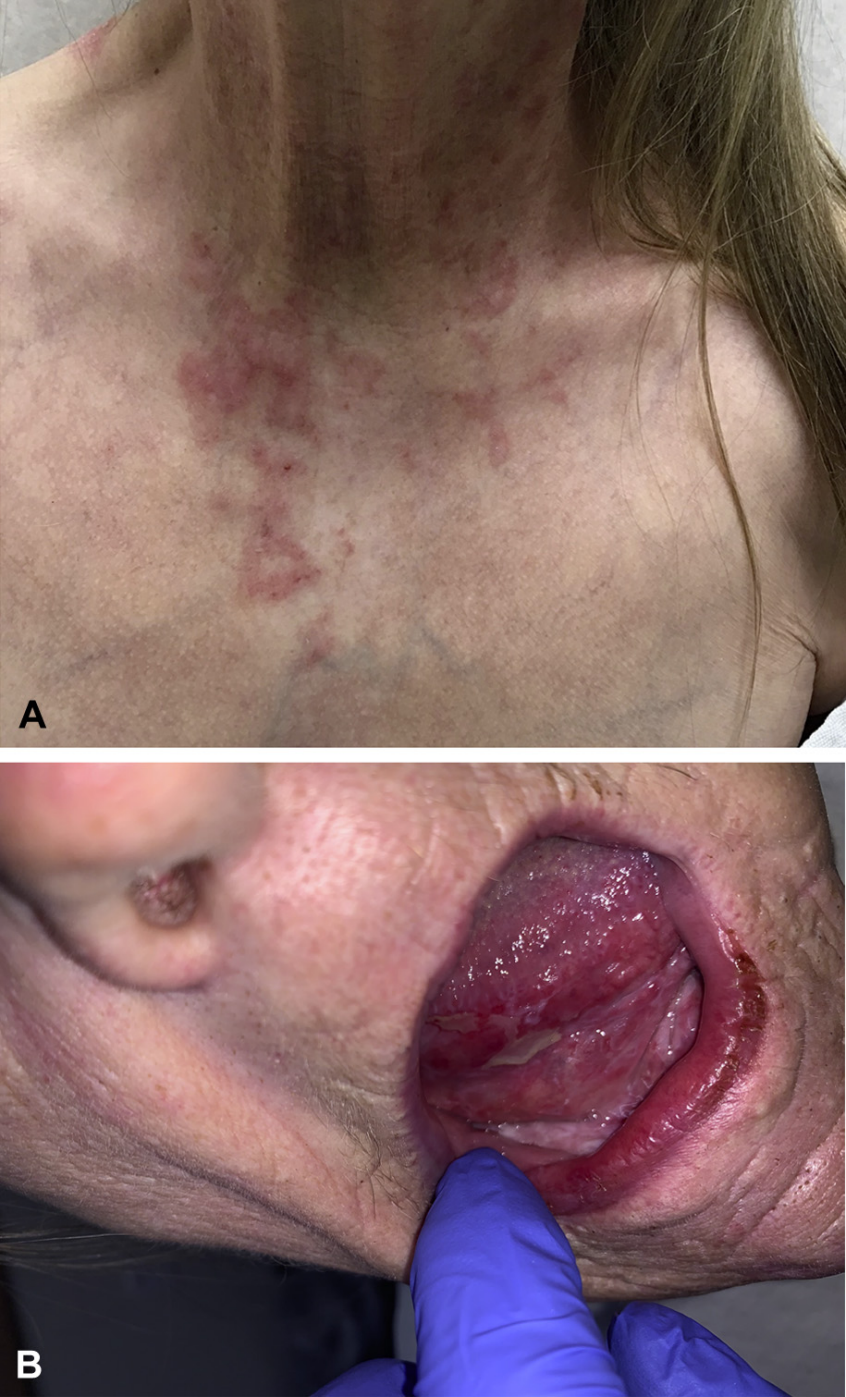
**A,** Numerous scattered erythematosus-to-pink papules and plaques with mild scale were seen throughout the neck, upper portion of the torso, and bilateral upper extremities. **B,** The extensive erosions and superficial ulcerations were noted through the oral mucosa, extending into the posterior aspect of the oropharynx.

Examination revealed extensive mucosal erosions and superficial ulcerations involving the tongue, buccal mucosa, hard/soft palate, and posterior oropharynx. In addition, the gingival mucosa demonstrated hypertrophy with a mild cobblestone appearance. Skin examination was notable for scattered erythematosus-to-pink arcuate papules and plaques with mild scale (Fig 1). No regional lymphadenopathy was appreciated on physical examination.

Extensive serologic and laboratory testing was performed, which revealed the following notable findings: Sodium, 125 mmol/L (reference range, 136-144 mmol/L); antinuclear antibodies (ANA), 1:320 (speckled pattern); SSA52-specific IgG, 44 AU/mL (reference range, 0-40 AU/mL), and SSA60-specific IgG 93 (reference range, 0-40 AU/mL). The following tests were notably negative or within the normal limits: Serum protein electrophoresis, HIV, hepatitis C, herpes simplex virus, Smith autoantibodies, histone autoantibodies, double-stranded DNA antibodies, and lactate dehydrogenase levels.

**Fig 2:**
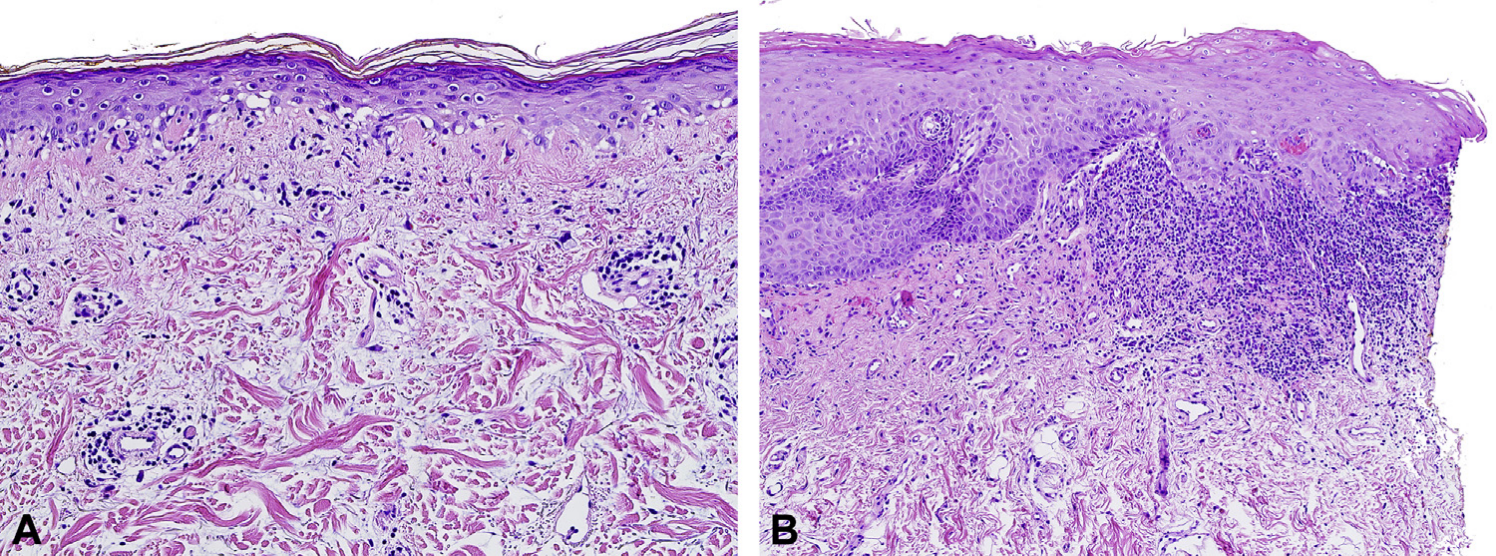
**A,** Punch biopsy from the upper portion of the back demonstrating an atrophic epidermis with vacuolar interface dermatitis, mild perivascular lymphocytic infiltrate with rare eosinophils, and increased dermal mucin deposition. **B,** Biopsy of the mucosal epithelium revealed hyperkeratosis, hypergranulosis, and a moderately dense, lichenoid infiltrate that focally obscured the basal layer of the epidermis, producing dyskeratotic keratinocytes and a few colloid bodies. (**A** and **B**, Hematoxylin-eosin stain; original manifications: **A,** 200×; **B,** 100×).

A skin biopsy of the upper portion of the back demonstrated a vacuolar interface dermatitis with perivascular lymphocytic infiltrate and increased dermal mucin, and an oral mucosal biopsy revealed a robust lichenoid interface dermatitis (Fig 2, *A* and *B*, respectively). Direct immunofluorescence microscopy showed grains of IgG around the basal and suprabasal epidermal cells. Indirect immunofluorescence microscopy was negative on monkey esophagus and rodent (mouse and rat) bladder substrates.

**Fig 3:**
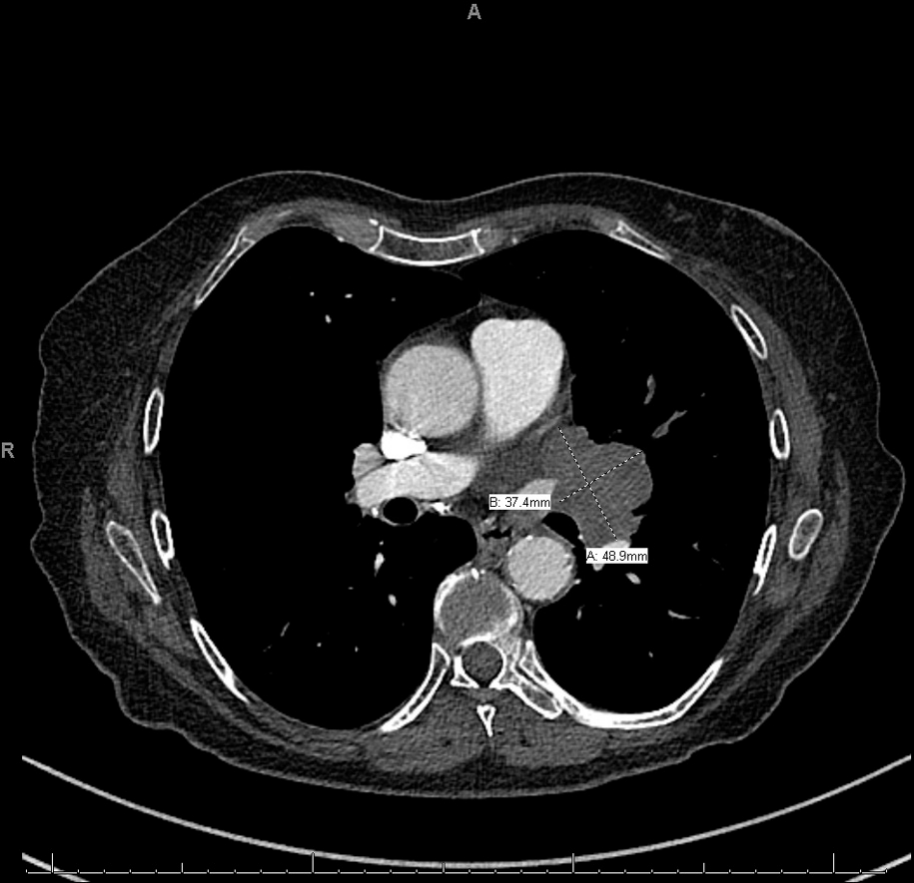
Chest computed tomography revealed a left perihilar mass measuring 4.9 × 3.7 cm with significant narrowing of the upper portion of the left lobe bronchus and pulmonary vein and narrowing of the lingual pulmonary artery. The mass was abutting the left main pulmonary artery and the left atrial appendage.

Chest X-ray followed by a high-resolution computed tomography scan demonstrated a 4.9-cm left perihilar mass that significantly compressed upon the upper portion of the left lobe bronchus, pulmonary vein, and artery (Fig 3). An endobronchial ultrasound-guided core needle biopsy was consistent with small-cell lung carcinoma.

Prior to the diagnosis of small-cell lung carcinoma, the patient had been placed on a 1 mg/kg-prednisone taper of 4 weeks with marked improvement, although her symptoms rebounded several weeks after the taper was completed. After her malignancy was established, prednisone (1 mg/kg) was restarted and tapered over 6 weeks, concurrent with chemotherapy and radiation.

The patient completed 4 cycles of carboplatin and etoposide and radiation therapy of 45 Gy in 30 Fractions. Following treatment, the patient reported no recurrence of cutaneous or oral disease.

## Discussion

SCLE is a photosensitive dermatosis that is part of the cutaneous lupus spectrum with a reported incidence of around 0.6 to 0.7 per 100,000 per year.^2^ It is commonly ANA- and Ro/SSA-positive, with the latter representing small ribonucleoproteins in the nucleus that translocate to the surface of keratinocytes upon UV exposure.^2^^,^^3^

Mucous membrane ulceration is an uncommon finding in SCLE, with 1 series recording 24% of their cohort. Yet, when present, mucosal involvement is more commonly associated with ANA, anti-DNA, Smith autoantibodies, and anti-ribonucleoprotein antibodies.^4^ Classically, SCLE has been associated with medications (drug-induced SCLE), and this possibility should be considered in every case. The most common culprit medications include proton pump inhibitors, thiazide diuretics, and antifungals.^5^^,^^6^ It should be noted that skin lesions of drug-induced SCLE are inseparable from those of non–drug related SCLE.^5^ In the present case, drug etiologies were thoroughly considered; however, the patient denied taking any prescription or over-the-counter medications prior to her mucocutaneous eruption.

With less than 20 cases reported in the literature, paraneoplastic SCLE is extremely rare. Although it has been associated with various malignancies, oroesophageal and lung carcinoma are most commonly reported, and the appearance of paraneoplastic SCLE may represent a late-stage manifestation.^3^^,^^7^^,^^8^ The pathogenesis of paraneoplastic SCLE is unknown but likely related to the expression of tumor antigens homologous to Ro antigen, with subsequent production of autoantibodies.^3^ For SCLE to be considered paraneoplastic, it should meet the Mclean criteria, wherein the dermatosis should develop after the malignancy but may be present before the diagnosis, and both dermatosis and malignancy follow a parallel course.^9^ Although rare, paraneoplastic SCLE is likely underappreciated and clinicians should have a high degree of suspicion for a paraneoplastic etiology, when patients present with atypical SCLE, such as cachexia or difficulty swallowing. We recommend performing basic laboratory (including lactate dehydrogenase test) and imaging studies (chest X-ray) in suspected cases.

The histologic findings in SCLE include vacuolar interface dermatitis with perivascular and periappendegeal lymphocytic infiltrate with extracellular mucin deposition.^2^ Distinctive staining patterns can be appreciated on direct immunofluorescence microscopy, where interepidermal deposition of IgG is present in the majority of patients.^10^ Remarkably, in our patient, this finding was appreciated on both cutaneous and oral mucous, helping to unify her clinical presentation.

Treatment for SCLE revolves around either identifying and removing suspicious medications or treating underlying malignancy, with concurrent use of topical, intralesional, or systemic steroids for acute management in severe cases. Antimalarial agents, such as hydroxychloroquine, can be employed to help with recalcitrant disease. Regression of paraneoplastic SCLE generally occurs with treatment of the underlying malignancy; however, few recalcitrant cases have been reported.^3^

SCLE is classically related to drug-induced etiologies; however, a paraneoplastic association should be considered in the appropriate clinical scenario (ie, culprit medications are absent). Therefore, management of paraneoplastic SCLE should be coordinated in a multidisciplinary approach with oncology, as regression is to be expected with the treatment of the underlying malignancy.

## Conflicts of interest

None disclosed.
